# Important Considerations for the Treatment of Patients with Diabetes Mellitus and Heart Failure from a Diabetologist’s Perspective: Lessons Learned from Cardiovascular Outcome Trials

**DOI:** 10.3390/ijerph17010155

**Published:** 2019-12-24

**Authors:** Chrysi Koliaki, Nicholas Katsilambros

**Affiliations:** 1 First Propaedeutic Department of Internal Medicine and Diabetes Center, National Kapodistrian University of Athens, Laiko University Hospital, 11527 Athens, Greece; nicholaskatsilambros@gmail.com; 2 Research Laboratory Christeas Hall, Medical School, National Kapodistrian University of Athens, 10679 Athens, Greece

**Keywords:** type 2 diabetes mellitus, heart failure, cardiovascular disease risk, cardiovascular outcome trials, glucose-lowering agents

## Abstract

Heart failure (HF) represents an important cardiovascular complication of type 2 diabetes mellitus (T2DM) associated with substantial morbidity and mortality, and is emphasized in recent cardiovascular outcome trials (CVOTs) as a critical outcome for patients with T2DM. Treatment of T2DM in patients with HF can be challenging, considering that these patients are usually elderly, frail and have extensive comorbidities, most importantly chronic kidney disease. The complexity of medical regimens, the high risk clinical characteristics of patients and the potential of HF therapies to interfere with glucose metabolism, and conversely the emerging potential of some antidiabetic agents to modulate HF outcomes, are only some of the challenges that need to be addressed in the framework of a team-based personalized approach. The presence of established HF or the high risk of developing HF in the future has influenced recent guideline recommendations and can guide therapeutic decision making. Metformin remains first-line treatment for overweight T2DM patients at moderate cardiovascular risk. Although not contraindicated, metformin is no longer considered as first-line therapy for patients with established HF or at risk for HF, since there is robust scientific evidence that treatment with other glucose-lowering agents such as sodium-glucose cotransporter 2 inhibitors (SGLT2i) should be prioritized in this population due to their strong and remarkably consistent beneficial effects on HF outcomes.

Diabetes mellitus (DM) and heart failure (HF) are recognized as global epidemics, since their prevalence is increasing worldwide with alarming rates [1]. DM and HF can occur concomitantly, and the risk of the one condition may independently increase the risk of the other [2]. In patients with HF, the prevalence of DM has been reported to range between 10 and 47% [3,4,5], and is even higher among hospitalized patients [6,7]. Conversely, in patients with DM, the prevalence of HF ranges between 9 and 22%, and is considerably higher in patients older than 60 years old [8,9]. When both DM and HF are present, the morbidity and mortality risks are substantially elevated, adverse patient outcomes are synergistically amplified and the healthcare costs are expanded [10,11]. Diabetic patients with HF have significantly worse cardiovascular (CV) outcomes than their non-diabetic counterparts [12], including an increased CV mortality by 50–90% [13], and in acute HF, an increased risk of in-hospital and 1-year all-cause death [14]. The serious prognostic implications of the coexistence of both conditions make any efforts for early prevention and treatment an ultimate clinical priority.

The relationship between DM and HF is bidirectional. On the one hand, DM increases more than 2-fold the risk of developing HF, both HF with reduced ejection fraction (HFrEF) and HF with preserved ejection fraction (HFpEF), and furthermore increases the risk of progression from asymptomatic left ventricular (LV) systolic dysfunction to symptomatic HF [4,15]. Vice versa, HF may increase the risk of developing type 2 diabetes mellitus (T2DM), considering that up to 60% of patients with HF are characterized by insulin resistance and metabolic dysregulation is both inherent and secondary to HF pathophysiology [16]. DM may promote the development and progression of HF both directly and indirectly via systemic, myocardial and cellular mechanisms [17]. Indirectly, DM can increase the risk of incident HF due to its coexistence with coronary heart disease (CHD), hypertension, atherogenic dyslipidemia and endothelial dysfunction, all of which are considered to be major predisposing factors for HF pathogenesis [17]. In addition to this indirect association, hyperglycemia, hyperinsulinemia and insulin resistance of the diabetic state exert direct negative effects on the myocardium, which have been collectively described with the term diabetic cardiomyopathy (DCM). This term was originally applied more than 40 years ago to define the systolic and/or diastolic dysfunction observed in patients with DM in the absence of other obvious causes of cardiomyopathy such as CHD, hypertension or valvulopathy [18]. Later on, the term DCM was broadened to integrate any kind of vulnerability of the diabetic myocardium to functional impairment. The major pathophysiological mechanisms implicated in the development of DCM include inflammation, oxidative stress, endoplasmic reticulum (ER) stress, insulin resistance, myocardial steatosis and lipotoxicity, maladaptive intracellular calcium homeostasis, altered myocardial substrate metabolism (shift from glucose to free fatty acid oxidation and vice versa), impaired mitochondrial bioenergetic efficiency, cardiac hypertrophy, cardiac fibrosis due to renin-angiotensin-aldosterone system (RAAS) activation and advanced glycation endproducts (AGEs) accumulation, impaired myocardial perfusion reserve and tissue hypoxia due to microvascular dysfunction, and autonomic neuropathy [19,20,21,22]. The fact that additional factors beyond glycemia may contribute to the increased risk of HF in patients with DM is substantiated by the observation that the elevated HF prevalence persists despite optimal glycemic control, and the normalization of blood glucose levels does not always restore CV risk to the non-diabetic levels [10].

In epidemiological terms, the association between DM and HF is stronger in women than men. This sex discrepancy, which remains still incompletely understood, was first observed in the Framingham Heart Study, where diabetic men presented with a 2-fold, while diabetic women presented with a 4-fold increased risk of HF after adjustment for age and other CV risk factors [23]. The same finding was further confirmed in a comprehensive systematic review and meta-analysis of 47 cohorts including more than 12 million Japanese diabetic patients, which demonstrated that both type 1 diabetes mellitus (T1DM) and T2DM are stronger risk factors for HF in women than in men [24]. Potential explanations for this gender-specific effect in the epidemiology of DM-associated HF include the higher prevalence of DCM in women, the stronger correlation of DM with CHD in women, the longer exposure of women to hyperglycemia in the prediabetic state, sex-specific differences in other CV risk factors, and finally the fact that diabetic men are more prone to premature death and thus will not survive to develop HF (survival bias) [25,26,27,28].

The concurrent presence of DM and HF and their shared pathophysiology underscore the need for an interdisciplinary collaborative management of both conditions [2]. Treatment of DM in patients with HF can be challenging, considering that patients with HF are usually elderly, frail and have extensive comorbidities, most importantly chronic kidney disease (CKD), which represents a major limitation to optimal drug dosing and hampers medication adherence due to possible drug-induced interactions and adverse effects. The complexity of medical regimens required for both conditions, the high risk clinical characteristics of patients and the well-established potential of HF therapies to interfere with glucose metabolism, and conversely the emerging potential of some antidiabetic agents to modulate HF outcomes, are only some of the clinical challenges that arise and need to be addressed in the framework of a team-based personalized approach [2]. The optimal glycemic targets for diabetic patients with HF should be individualized to reflect life expectancy and comorbidity burden (HF severity and DM complications), and the benefits expected from glucose-lowering therapies should be always balanced with the risks of hypoglycemia, polypharmacy and treatment costs [2]. 

The multiple challenges of treating patients with DM and cardiovascular disease (CVD), especially HF, have been addressed in clinical practice guidelines and position statements issued by international scientific societies such as the European Society of Cardiology (ESC), the European Association for the Study of Diabetes (EASD), the American Heart Association (AHA) and the Heart Failure Society of America (HFSA), which aim to provide evidence-based guidance on how to optimally treat patients with DM and CV complications [2,29]. In the updated version of these guidelines, patients with DM are stratified based on their CVD risk into medium, high and very high risk patients [29]. Young patients (<35 years for T1DM; <50 years for T2DM) with a short duration of DM (<10 years) without major CV risk factors are considered to be at medium risk. Patients with a longer DM duration (>10 years) and at least one major CV risk factor without signs of target organ damage are placed at high risk, and patients with established CVD or at least 3 major CV risk factors or evidence of target organ damage or long-standing T1DM (>20 years) are classified as very high risk. Based on the above stratification, the selection of the most appropriate antidiabetic agent should be tailored depending on the presence of established CVD or CVD risk [29].

Over the past 5–6 years, the landscape of T2DM treatment has been revolutionized by the introduction of novel glucose-lowering agents, which -for the first time in the history of DM pharmacotherapy- have shown the potential to improve CV outcomes in patients with T2DM. The so-called Cardiovascular Outcome Trials (CVOTs), namely randomized clinical trials originally designed to fulfil the Food and Drug Administration (FDA) requirement to assess CV safety of antidiabetic agents vs. placebo, have recently provided an overwhelming body of evidence suggesting that two specific antidiabetic drug classes exert cardioprotective effects in diabetic patients with CVD or at risk for CVD. These drug classes comprise glucagon-like peptide 1 receptor agonists (GLP1RAs) and sodium-glucose cotransporter 2 inhibitors (SGLT2i). The former have been primarily associated with a reduction in atherosclerosis-related outcomes and major adverse cardiovascular events (MACEs), whereas the latter have been linked to an improvement in HF-related endpoints. To date, several CVOTs have been published and influenced dramatically clinical guidelines and treatment algorithms: 4 for dipeptidyl peptidase 4 inhibitors (DPP4i) (sitagliptin, saxagliptin, linagliptin, alogliptin) [30,31,32,33], 7 for GLP1RAs (exenatide, lixisenatide, albiglutide, liraglutide, oral and injectable semaglutide, dulaglutide) [34,35,36,37,38,39,40] and 3 for SGLT2i (empagliflozin, canagliflozin, dapagliflozin) [41,42,43]. The major take-home messages derived from the recent CVOTs comprise the following:

(1) Most DPP4i have neutral effects on MACEs and HF outcomes with the exception of saxagliptin, which has been shown to increase significantly the risk of HF hospitalization in high risk patients [31]. Although meta-analyses and real-world observational studies found no significant risk of HF hospitalization with DPP4i vs. placebo [44,45], a recent network meta-analysis reported a higher risk of HF with DPP4i compared with other antidiabetic drug classes [46]. Considering the lack of evidence that DPP4i provide any CV benefit and the compelling evidence that some of them can even increase HF hospitalization risk, DPP4i are not the most appropriate choice for diabetic patients with pre-existing HF or at risk for developing HF (i.e., older patients, obese, long DM duration or CKD);
(2) GLP1RAs have neutral impact on HF hospitalization, but some of them (liraglutide, semaglutide, dulaglutide) reduce the risk of MACEs, including CV mortality, in diabetic patients with CVD or high CVD risk [34,35,40]. GLP1RAs are recommended for diabetic patients with CVD or at high/very high risk for CVD in order to reduce CV events. Their use in patients with HF or at risk for HF is questionable considering the disappointing results of two small randomized clinical trials showing a trend for increased HF hospitalization and worse outcomes with liraglutide in patients with HFrEF [47,48];
(3) SGLT2i are the first antidiabetic drug class which has demonstrated the potential to reduce mortality, MACEs and HF hospitalization in diabetic patients at high CVD risk, regardless of the history of previous HF [41,49,50]. Their beneficial effects are independent of glycemic control and occur too early to be attributed to the concomitant weight reduction. Based on these findings, SGLT2i are highly recommended as first-line treatment for diabetic patients with established HF or at risk to develop HF, in order to reduce hospitalization and improve prognosis. The expected benefits should be critically balanced against potential risks of genitourinary infections, euglycemic diabetic ketoacidosis and lower limb amputations and fractures, the latter related only to canagliflozin but not confirmed in recent studies [51]. The major mechanisms mediating the strong cardioprotective effects of SGLT2i involve their modest diuretic and natriuretic effects, weight loss, reduction of blood pressure, anti-oxidant and anti-inflammatory properties, amelioration of renal congestion, reduction of plasma volume without subsequent neurohormonal activation, increased supply of ketones to cardiomyocytes and improved mitochondrial bioenergetics [52,53,54,55,56].

Interestingly, both GLP1RAs (liraglutide and semaglutide) and SGLT2i (dapagliflozin, canagliflozin, empagliflozin) have shown additional renoprotective effects in patients with DM. The major CVOTs of these agents have studied renal function as a secondary outcome and have shown that compared with placebo, these drugs are able to delay the progression of CKD and reduce clinically significant renal events [34,35,41,42,43]. Canagliflozin, in particular, has shown the potential to reduce by 30% the primary renal outcome of end-stage renal disease, doubling of serum creatinine or renal death in patients with T2DM, as well as attenuate the slope of progressive renal function decline [57]. Furthermore, recent compelling evidence suggests that the SGLT2i dapagliflozin is able to reduce the composite outcome of HF worsening or CV death in patients with HFrEF of moderate severity, regardless of the presence of DM [58]. These data highlight the potential of SGLT2i to serve as HF treatment even in patients without DM, and need to be corroborated with additional studies.

Despite the progress summarized above, a large number of unanswered questions and knowledge gaps remain still under investigation and should be thoroughly examined in the future:

- Is DCM reversible and which other mechanisms -beyond the already identified ones- may contribute to the pathophysiology of this entity?
- Which mechanisms could explain the divergent evidence on the association of DPP4i with HF risk showing an increased risk with saxagliptin but no increased risk with the other gliptins?
- Are the effects of GLP1RAs on HF outcomes neutral or potentially harmful? In case of adverse effects, are they attributable to the modest increase in heart rate induced by GLP1RAs, or are other mechanisms involved?
- Are SGLT2i equally safe and effective in patients with HFrEF and HFpEF?
- What is the role of SGLT2i as a stand-alone HF treatment in non-diabetic patients (i.e., patients with HF and prediabetes)?
- What are the exact mechanistic/hemodynamic effects of SGLT2i on LV remodelling and myocardial injury beyond osmotic diuresis?
- Which are the optimal glycemic targets for diabetic patients with different severity of HF?
- What is the optimal antidiabetic agent for patients with HF and advanced CKD? This issue is particularly relevant considering that SGLT2i are on the one hand able to confer renoprotection, but on the other hand are not indicated for use in patients with an estimated glomerular filtration rate (eGFR) below 30 mL/min/1.73 m^2^.
- Is the use of novel antidiabetic therapies such as SGLT2i expected to change the epidemiology of DM-associated HF?

Future research in the field of cardiovascular diabetology should try to resolve these issues and provide definitive answers. Furthermore, it is important that future trials integrate non-invasive cardiac imaging (i.e., echocardiography, cardiac magnetic resonance imaging) in order to establish novel imaging biomarkers, which would help better characterize the structural and functional abnormalities of the diabetic failing heart and facilitate monitoring of treatment effects [59].

In conclusion, HF represents an important CV complication of DM associated with substantial morbidity and mortality, and is emphasized in recent CVOTs as a critical outcome for patients with DM. The presence of HF or the high risk for developing HF in the future has influenced recent guideline recommendations and can guide therapeutic decision making. Metformin remains first-line treatment for overweight T2DM patients without CVD and at moderate CVD risk. Although not contraindicated at any stage of HF, metformin is no longer considered as first-line therapy for diabetic patients with established HF or at risk for HF, since there is robust scientific evidence that treatment with other glucose-lowering agents such as SGLT2i should be prioritized in this population due to their strong beneficial effects on HF outcomes.
